# Supplementary material for: Epidemiology, clinical characteristics, household transmission, and lethality of severe acute respiratory syndrome coronavirus-2 infection among healthcare workers in Ontario, Canada
Source: PLoS One. 2020 Dec 28;15(12):e0244477. doi: 10.1371/journal.pone.0244477 (PMC7769426; doi:10.1371/journal.pone.0244477)
Supplement: Attachment — Submitted filename: Response to reviewers_NOV26_2020.docx [file pone.0244477.s001.docx]

Dear Dr. Manzoli,

We appreciate the constructive feedback provided from yourself and the reviewer. The manuscript has been substantially revised based on this including a multivariable model that evaluates the risk of death between healthcare workers and non-healthcare workers.

Public Health Ontario (PHO) cannot disclose the underlying data. Doing so would compromise individual privacy contrary to PHO’s ethical and legal obligations. Restricted access to the data may be available under conditions prescribed by the Ontario Personal Health Information Protection Act, 2004, the Ontario Freedom of Information and Protection of Privacy Act, the Tri-Council Policy Statement: Ethical Conduct for Research Involving Humans (TCPS 2 (2018)), and PHO privacy and ethics policies. Data are available for researchers who meet PHO’s criteria for access to confidential data. Information about PHO’s data access request process is available on-line at <https://www.publichealthontario.ca/en/data-and-analysis/using-data/data-requests>.

Sincerely,

Kevin Schwartz, MD MSc

On behalf of the co-authors

R2 – Editor comments

1. We note that the specific dataset you used in your study may be third party data (i.e. data not owned or collected by the authors) from Ontario’s Ministry of Health.

Before we proceed, please clarify if the modified dataset you mentioned in your Data Availability statement is equivalent to your 'minimal dataset', which PLOS defines as consisting of the data set used to reach the conclusions drawn in the manuscript with related metadata and methods, and any additional data required to replicate the reported study findings in their entirety. This includes:

a) The values behind the means, standard deviations and other measures reported;

b) The values used to build graphs;

c) The points extracted from images for analysis.

**Response**: After reviewing your comments with our research department our response has been modified regarding data availability:

Public Health Ontario (PHO) cannot disclose the underlying data. Doing so would compromise individual privacy contrary to PHO’s ethical and legal obligations. Restricted access to the data may be available under conditions prescribed by the Ontario Personal Health Information Protection Act, 2004, the Ontario Freedom of Information and Protection of Privacy Act, the Tri-Council Policy Statement: Ethical Conduct for Research Involving Humans (TCPS 2 (2018)), and PHO privacy and ethics policies. Data are available for researchers who meet PHO’s criteria for access to confidential data. Information about PHO’s data access request process is available on-line at <https://www.publichealthontario.ca/en/data-and-analysis/using-data/data-requests>.

2. We note that you mention Fig 1 is not copyrighted.

Before we proceed, please clarify if the authors created this map themselves or retrieved it from another source.

If you retrieved it from another source, please provide the name of the source.

**Response**: The authors created the map.

3. Thank you for the clarification about the activities described not being research.

Please provide a waiver or letter from the PHO Ethics Review Board stating that this study was not considered research and explaining the reasons as to why.

The letter or waiver should also state whether the need for informed consent was waived.

In addition, please indicate whether data was anonymized before or after the researchers received it.

**Response**: The data is used for public health purposes and therefore the analyst has access to patient identifiers. The remainder of the research team only has access to anonymized and aggregated data. Requested letter provided from PHO’s Ethics Review Board has been included with the resubmission.

R1

1.

Throughout the manuscript, COVID-19 cases (disease) have been confused with SARS-CoV-2 infections. This is clear because also the asymptomatic subjects have been included among the "cases". This is plain wrong, misleading for the reader, and definitely requires to be corrected everywhere.

**Response: This has been corrected throughout.**

2.

In the methodology, it is written that all COVID-19 cases (which is referred to SARS-CoV-2 infections) have been captured by Public Health Ontario. Later in the manuscript, it is written repeatedly that many infections have likely been lost. Consistently, the authors should refer to their sample as "all the SARS-CoV-2 infections that have been diagnosed".

**Response: This has been corrected in the methods and throughout.**

3.

No details have been provided on the "natural language processing" algorithm, that has been used to identify the household transmission (an essential part of the study). Was the household referred solely to those residing in the same house, or also to the neighbours? This clearly needs to be clarified.

**Response: Further details of the NLP algorithm added (page 6-7 line 127-131): “The algorithm used Python’s sklearn library. Address text was broken down into short segments (N-grams) with a term-frequency inverse document frequency matrix. The closest proximity match within the matrix was returned and validated using checks for numerical portions of the address field, including suite number if available. The algorithm matched those within the same household or apartment unit, and not to neighbours.”**

4.

Mortality has been sometimes confused with lethality. Please correct it everywhere.

**Response: The term “mortality” has been changed to “death” or “lethality” throughout.**

5.

Insufficient details have been provided on the definition of healthcare workers. It is only written that they are "caring for patients". Does this definition include also cohabiting caregivers who are not employees of the National healthcare system? If so, how many? Given that only 22.6% of the healthcare workers were physicians or nurses, more information on the remaining 77.4% is definitively needed. In the manuscript, the only detail is given in the footnote b of Table 1, where it is written that "at least 708 (21.6%) were personal support workers identified through free text searching". First, what does "at least mean? Second, what about the remaining 1968? Who are they? This is an essential information that is needed to understand the adopted definition of healthcare worker.

**Response: Additional details on the definition of HCW has been added (Page 5 line 100-104). “A HCW was defined as an individual who self-reported to have an occupation involving caring for patients including (but not limited to) doctors, nurses, dentists, dental hygienists, midwives, other medical technicians, personal support workers, respiratory therapists, and first responders. The database has fields for doctor and nurse, however all other HCWs were recorded as “other” with the opportunity for free text.”**

6.

A number of comorbidities have been listed as available, but these data have not been reported and, even more importantly, they have not been used to perform multivariate analyses, which are essential to make any meaningful comparison between the risk of death of healthcare workers and the rest of the population. This point needs to be clarified and data, if really available, provided for both healthcare workers and the rest of the population. Also, if these data are available, a multivariate analysis predicting the risk of death of healthcare workers versus others should be performed and would be the most interesting part of the study (for the limitations that will be mentioned later).

**Response: A multivariable model evaluating the risk of death between HCWs and non-HCWs has now been added as suggested (Table 4). In addition, the comorbidity data has been expanded and utilized for this analysis as suggested.**

7.

The information on nosocomial transmission is missing for 76.9% of the healthcare workers, and 87.5% of the population. Despite this clear, huge bias, the authors extensively discuss these data, just briefly mentioning the missing data issue. With such a large amount of missing data, any discussion of the result should be avoided. The risk of bias of these data is too large to be meaningful in any way, and they can also be misleading. All analyses on nosocomial infections can be shown but briefly mentioned in the Results as merely indicative. Do not mention them in the Discussion (if not in the limitations), Conclusions or Abstract.

**Response: Reference to the nosocomial data in discussion, conclusion, and abstract has been removed (aside from the noted limitation on line 300).**

8.

Both the Results and the Discussion need to be improved. In the Results there some confusion, the most important data, those on the comparisons of the infection and mortality rates, have been reported after some trivial data (as the difference in the proportion of asymptomatic cases, a clear example of a statistically significant yet meaningless finding - 8.1% versus 7.0%).

The Discussion is redundant, and does not follow a clear reasoning. There also are some speculations (page 8, lines 177-178 on Ontario), some sentences that are to placed into an Introduction (page 10, lines 212-215) and, most importantly, it is totally unclear how the conclusions of the authors "we feel that data highlight the importance of community and household risk for HCWs, maintaining physicial distancing from colleagues and utilizing addition PPI..."

First, all the conclusions were known before the study. Second, and most importantly, it is not explained why the results of the study lead the authors to "feel" this way. In this study, nosocomial infections (with all the limitations above reported) were as low as 3.6%, and household transmissions were also relatively low (9.8%; lower than many other non-cited studies). It is unclear why these findings should bring us to "emphasize the protection policies of healthcare workers". This definitively needs explanation.

Also, as briefly mentioned before, the discussion on the potential explanations for the observed difference in lethality (not mortality) should be based upon multivariate analyses. Otherwise, it is speculative.

**Response: The results and discussion has been updated with the new results and substantially re-written with sections removed and/or revised as suggested. We have added the multivariable model with death as the outcome (table 4) and added discussion on this. We hope you will find the discussion more organized and supported by the data presented.**

9.

Page 10, lines 217-220: how many children were there to be infected? This point needs clarification.

**Response: Denominator for household contacts are not known. As stated in the methods this data is only positive cases with SARS-CoV-2 infection. This has been added to the limitations (line 308)**

10.

Limitations need to be expanded, including the absence of a multivariate analysis, the huge missing data (not only for nosocomial infections, but also the symptoms were missing for almost 40% of the population), and the unavoidable underestimation of the SARS-CoV-2 infection in the total population.

**Response: A multivariable model was added and reference to nosocomial data removed due to the large amount of missing data. With updating the results missing symptom data has been greatly reduced. The missing data has been retained in the multivariable model as its own covariate. Further discussion throughout the manuscript has been added related to the underestimation of general population cases.**

11.

The abstract is the weakest part of the manuscript and needs to be extensively revised. First, the comparison is not stated, it is repeatedly stated that "HCWs were more likely...", but it is not clear "than who?".

**Response: The abstract has been re-written and updated with the new results.**

12.

No definition of acquisition is provided, nosocomial infections need to be cut, mortality is lethality, COVID-19 is SARS-CoV-2 infection, it is not clear whether the 9.8% of probable secondary household transmissions are cases or episodes, low numbers are low percentages, the conclusion is unsubstantiated, and other issues as above.

**Response: Acquisition definition is provided in the methods lines 131-135 (household case with symptom onset data 2+ days prior to the HCW). As a sensitivity analysis we used +/-4 days. Reference to nosocomial variable has been removed from the results text and discussion (aside from the limitations). Mortality has been changed to death or lethality throughout as suggested. COVID-19 has been changed to SARS-CoV-2 infection as suggested throughout. The household transmission data represents new infections (see methods lines 131-135). The results have been substantially revised to not discuss less important findings. The discussion and conclusion (including in the abstract) have been substantially revised and re-written based on the feedback provided and updated results.**

Minor issues

13.

References 4-6 could be updated.

**Response: Newer references have been added in addition to these from earlier in the pandemic.**

14.

Page 3, lines 47-48: it is unclear whether this point is related to the aim of the study.

**Response: This line has been removed.**

Please submit your revised manuscript by Sep 21 2020 11:59PM. If you will need more time than this to complete your revisions, please reply to this message or contact the journal office at [plosone@plos.org](mailto:plosone@plos.org). When you're ready to submit your revision, log on to <https://www.editorialmanager.com/pone/> and select the 'Submissions Needing Revision' folder to locate your manuscript file.

Please include the following items when submitting your revised manuscript:

- A rebuttal letter that responds to each point raised by the academic editor and reviewer(s). You should upload this letter as a separate file labeled 'Response to Reviewers'.
- A marked-up copy of your manuscript that highlights changes made to the original version. You should upload this as a separate file labeled 'Revised Manuscript with Track Changes'.
- An unmarked version of your revised paper without tracked changes. You should upload this as a separate file labeled 'Manuscript'.

If you would like to make changes to your financial disclosure, please include your updated statement in your cover letter. Guidelines for resubmitting your figure files are available below the reviewer comments at the end of this letter.

If applicable, we recommend that you deposit your laboratory protocols in protocols.io to enhance the reproducibility of your results. Protocols.io assigns your protocol its own identifier (DOI) so that it can be cited independently in the future. For instructions see: [http://journals.plos.org/plosone/s/submission-guidelines#loc-laboratory-protocols](https://can01.safelinks.protection.outlook.com/?url=http%3A%2F%2Fjournals.plos.org%2Fplosone%2Fs%2Fsubmission-guidelines%23loc-laboratory-protocols&data=02%7C01%7Ckevin.schwartz%40oahpp.ca%7C31f50604f829426357d908d83afbff1b%7Ccddc1229ac2a4b97b78a0e5cacb5865c%7C0%7C0%7C637324201280511212&sdata=KOOgogBtp%2FNZKzgGAEl6PoyStMNBxEpNozufKQTjiOA%3D&reserved=0)

We look forward to receiving your revised manuscript.

Kind regards,

Lamberto Manzoli, M.D., M.P.H.
Academic Editor
PLOS ONE

Journal Requirements:

When submitting your revision, we need you to address these additional requirements.

1. Please ensure that your manuscript meets PLOS ONE's style requirements, including those for file naming. The PLOS ONE style templates can be found at

<https://journals.plos.org/plosone/s/file?id=wjVg/PLOSOne_formatting_sample_main_body.pdf> and

<https://journals.plos.org/plosone/s/file?id=ba62/PLOSOne_formatting_sample_title_authors_affiliations.pdf>

2. In your ethics statement in the Methods section and in the online submission form, please clarify whether all data were fully anonymized before you accessed them and/or whether the IRB or ethics committee waived the requirement for informed consent.

**Response: This statement was added (Page 4 line 70).**

3. In your Data Availability statement, you have not specified where the minimal data set underlying the results described in your manuscript can be found. PLOS defines a study's minimal data set as the underlying data used to reach the conclusions drawn in the manuscript and any additional data required to replicate the reported study findings in their entirety. All PLOS journals require that the minimal data set be made fully available. For more information about our data policy, please see <http://journals.plos.org/plosone/s/data-availability>.

Upon re-submitting your revised manuscript, please upload your study’s minimal underlying data set as either Supporting Information files or to a stable, public repository and include the relevant URLs, DOIs, or accession numbers within your revised cover letter. For a list of acceptable repositories, please see <http://journals.plos.org/plosone/s/data-availability#loc-recommended-repositories>. Any potentially identifying patient information must be fully anonymized.

Important: If there are ethical or legal restrictions to sharing your data publicly, please explain these restrictions in detail. Please see our guidelines for more information on what we consider unacceptable restrictions to publicly sharing data: <http://journals.plos.org/plosone/s/data-availability#loc-unacceptable-data-access-restrictions>. Note that it is not acceptable for the authors to be the sole named individuals responsible for ensuring data access.

We will update your Data Availability statement to reflect the information you provide in your cover letter.

**Response**: Public Health Ontario (PHO) cannot disclose the underlying data. Doing so would compromise individual privacy contrary to PHO’s ethical and legal obligations. Restricted access to the data may be available under conditions prescribed by the Ontario Personal Health Information Protection Act, 2004, the Ontario Freedom of Information and Protection of Privacy Act, the Tri-Council Policy Statement: Ethical Conduct for Research Involving Humans (TCPS 2 (2018)), and PHO privacy and ethics policies. Data are available for researchers who meet PHO’s criteria for access to confidential data. Information about PHO’s data access request process is available on-line at <https://www.publichealthontario.ca/en/data-and-analysis/using-data/data-requests>.

4. We note that Figure 1 in your submission contain map images which may be copyrighted. All PLOS content is published under the Creative Commons Attribution License (CC BY 4.0), which means that the manuscript, images, and Supporting Information files will be freely available online, and any third party is permitted to access, download, copy, distribute, and use these materials in any way, even commercially, with proper attribution. For these reasons, we cannot publish previously copyrighted maps or satellite images created using proprietary data, such as Google software (Google Maps, Street View, and Earth). For more information, see our copyright guidelines: <http://journals.plos.org/plosone/s/licenses-and-copyright>.

We require you to either (1) present written permission from the copyright holder to publish these figures specifically under the CC BY 4.0 license, or (2) remove the figures from your submission:

4.1.    You may seek permission from the original copyright holder of Figure 1 to publish the content specifically under the CC BY 4.0 license.

We recommend that you contact the original copyright holder with the Content Permission Form (<http://journals.plos.org/plosone/s/file?id=7c09/content-permission-form.pdf>) and the following text:

“I request permission for the open-access journal PLOS ONE to publish XXX under the Creative Commons Attribution License (CCAL) CC BY 4.0 (<http://creativecommons.org/licenses/by/4.0/>). Please be aware that this license allows unrestricted use and distribution, even commercially, by third parties. Please reply and provide explicit written permission to publish XXX under a CC BY license and complete the attached form.”

Please upload the completed Content Permission Form or other proof of granted permissions as an "Other" file with your submission.

In the figure caption of the copyrighted figure, please include the following text: “Reprinted from [ref] under a CC BY license, with permission from [name of publisher], original copyright [original copyright year].”

4.2.    If you are unable to obtain permission from the original copyright holder to publish these figures under the CC BY 4.0 license or if the copyright holder’s requirements are incompatible with the CC BY 4.0 license, please either i) remove the figure or ii) supply a replacement figure that complies with the CC BY 4.0 license. Please check copyright information on all replacement figures and update the figure caption with source information. If applicable, please specify in the figure caption text when a figure is similar but not identical to the original image and is therefore for illustrative purposes only.

The following resources for replacing copyrighted map figures may be helpful:

USGS National Map Viewer (public domain): <http://viewer.nationalmap.gov/viewer/>

The Gateway to Astronaut Photography of Earth (public domain): <http://eol.jsc.nasa.gov/sseop/clickmap/>

Maps at the CIA (public domain): <https://www.cia.gov/library/publications/the-world-factbook/index.html> and <https://www.cia.gov/library/publications/cia-maps-publications/index.html>

NASA Earth Observatory (public domain): <http://earthobservatory.nasa.gov/>

Landsat: <http://landsat.visibleearth.nasa.gov/>

USGS EROS (Earth Resources Observatory and Science (EROS) Center) (public domain): [http://eros.usgs.gov/#](http://eros.usgs.gov/)

Natural Earth (public domain): <http://www.naturalearthdata.com/>

**Response: The figures are not copyrighted and were created by the authors.**


[Note: HTML markup is below. Please do not edit.]

Reviewers' comments:

Reviewer's Responses to Questions

**Comments to the Author**

1. Is the manuscript technically sound, and do the data support the conclusions?

The manuscript must describe a technically sound piece of scientific research with data that supports the conclusions. Experiments must have been conducted rigorously, with appropriate controls, replication, and sample sizes. The conclusions must be drawn appropriately based on the data presented.

Reviewer #1: Yes

2. Has the statistical analysis been performed appropriately and rigorously?

Reviewer #1: Yes

3. Have the authors made all data underlying the findings in their manuscript fully available?

The [PLOS Data policy](https://can01.safelinks.protection.outlook.com/?url=http%3A%2F%2Fwww.plosone.org%2Fstatic%2Fpolicies.action%23sharing&data=02%7C01%7Ckevin.schwartz%40oahpp.ca%7C31f50604f829426357d908d83afbff1b%7Ccddc1229ac2a4b97b78a0e5cacb5865c%7C0%7C0%7C637324201280521206&sdata=EtUlfnhF8%2F0ajFGg8G2GvoGfCP49tvE9%2F2l43r4%2BcaA%3D&reserved=0) requires authors to make all data underlying the findings described in their manuscript fully available without restriction, with rare exception (please refer to the Data Availability Statement in the manuscript PDF file). The data should be provided as part of the manuscript or its supporting information, or deposited to a public repository. For example, in addition to summary statistics, the data points behind means, medians and variance measures should be available. If there are restrictions on publicly sharing data—e.g. participant privacy or use of data from a third party—those must be specified.

Reviewer #1: Yes

4. Is the manuscript presented in an intelligible fashion and written in standard English?

PLOS ONE does not copyedit accepted manuscripts, so the language in submitted articles must be clear, correct, and unambiguous. Any typographical or grammatical errors should be corrected at revision, so please note any specific errors here.

Reviewer #1: Yes

5. Review Comments to the Author

Please use the space provided to explain your answers to the questions above. You may also include additional comments for the author, including concerns about dual publication, research ethics, or publication ethics. (Please upload your review as an attachment if it exceeds 20,000 characters)

Reviewer #1: I have only a few minor suggestions:
p. 2 line 29 Given that the majority of HCW are ‘other’, it would be helpful to include at least the example of the largest group in the abstract. If these are personal support workers, I think this is important to put in the abstract.

**Response: We have further explained the definition and data available to describe the type of HCW (page 5 line 100-104): “A HCW was defined as an individual who self-reported to have an occupation involving caring for patients including (but not limited to) doctor, nurse, dentist, dental hygienist, midwife, other medical technicians, personal support worker, respiratory therapist, and first responder. The database has fields for doctor and nurse, however all other HCWs were recorded as “other” with the opportunity for free text.” From the data we cannot be certain on the precise numbers within the “other” category. We performed free text searching for PSW to attempt to delineate this further but we prefer not to emphasize this since it is possible we are missing PSWs since it relies on free text entry.**

p. 3 line 49 suggest revising to read “reported from a single centre has ranged”

**Response: corrected as suggested.**

p. 4 line 75. Many people will not know what a Crown corporation is. Can you use a more descriptive or generic term?

**Response: Changed crown to government**

p. 4 line 81 insert ‘and’ before comorbidities

**Response: Added**

p. 5 line 100. Is there any way or sense of how multiple occupancy addresses (apartment buildings were included and how they were handled in the data analysis?

**Response: Further details on the NLP algorithm was added including the incorporation of apartment suite numbers (page 6 line 126-131): “The NLP algorithm used Python’s sklearn library. Address text was broken down into short segments with a term-frequency inverse document frequency matrix (TF-IDF). The closest proximity match within the TF-IDF matrix was returned and validated using checks for numerical portions of the address field, including suite number if available.”**

p. 7 and other pages. Can you clarify if you are defining nosocomial and acquired in a hospital or acquired in the place the HCW works/from a patient. As most infections are in personal support workers, I found this confusing and the discuss about this on p.8 line 178 to the end of the paragraph took me several readings to understand and I am still not sure I am following correctly. Is all of this discussion about the fact that it is hard to be sure how HCWs were infected, or is it more complex?

**Response: Based on the editors comments discussion of this variable has been substantially de-emphasized based on the degree of missing data.**

6. PLOS authors have the option to publish the peer review history of their article ([what does this mean?](https://can01.safelinks.protection.outlook.com/?url=https%3A%2F%2Fjournals.plos.org%2Fplosone%2Fs%2Feditorial-and-peer-review-process%23loc-peer-review-history&data=02%7C01%7Ckevin.schwartz%40oahpp.ca%7C31f50604f829426357d908d83afbff1b%7Ccddc1229ac2a4b97b78a0e5cacb5865c%7C0%7C0%7C637324201280521206&sdata=Iju8Gu%2B6sdLN84r73H34or0BVmrJMTTQJfOuztZ%2FzJM%3D&reserved=0)). If published, this will include your full peer review and any attached files.


If you choose “no”, your identity will remain anonymous but your review may still be made public.


**Do you want your identity to be public for this peer review?** For information about this choice, including consent withdrawal, please see our [Privacy Policy](https://can01.safelinks.protection.outlook.com/?url=https%3A%2F%2Fwww.plos.org%2Fprivacy-policy&data=02%7C01%7Ckevin.schwartz%40oahpp.ca%7C31f50604f829426357d908d83afbff1b%7Ccddc1229ac2a4b97b78a0e5cacb5865c%7C0%7C0%7C637324201280531204&sdata=xB8Q8Ywv1RRTUCtEbldhQIPiko4QcvwyCqkSYSrqfpM%3D&reserved=0).

Reviewer #1: **Yes:** Annette M. Totten

[NOTE: If reviewer comments were submitted as an attachment file, they will be attached to this email and accessible via the submission site. Please log into your account, locate the manuscript record, and check for the action link "View Attachments". If this link does not appear, there are no attachment files.]

While revising your submission, please upload your figure files to the Preflight Analysis and Conversion Engine (PACE) digital diagnostic tool, [https://pacev2.apexcovantage.com/](https://can01.safelinks.protection.outlook.com/?url=https%3A%2F%2Fpacev2.apexcovantage.com%2F&data=02%7C01%7Ckevin.schwartz%40oahpp.ca%7C31f50604f829426357d908d83afbff1b%7Ccddc1229ac2a4b97b78a0e5cacb5865c%7C0%7C1%7C637324201280531204&sdata=jKbd62hmoA4m6tWg112UGFfnVgeEqC86IfRDzDTb9Ys%3D&reserved=0). PACE helps ensure that figures meet PLOS requirements. To use PACE, you must first register as a user. Registration is free. Then, login and navigate to the UPLOAD tab, where you will find detailed instructions on how to use the tool. If you encounter any issues or have any questions when using PACE, please email PLOS at [figures@plos.org](mailto:figures@plos.org). Please note that Supporting Information files do not need this step.
